# Calciphylaxis: A Case Report and Review of the Role of Sodium Thiosulphate and Other Treatment Modalities

**DOI:** 10.7759/cureus.74859

**Published:** 2024-11-30

**Authors:** Marcus Dawson, Sonya Limbu, Jeremy Chan, Ojone Usman, Chintana Galahitiyawa

**Affiliations:** 1 Internal Medicine, Colchester Hospital, Colchester, GBR; 2 Medicine, Colchester Hospital, Colchester, GBR; 3 Nephrology, Colchester Hospital, Colchester, GBR

**Keywords:** calciphylaxis, end-stage renal disease (esrd), haemodialysis, secondary hyperparathyroidism, sodium thiosulphate

## Abstract

Calciphylaxis is a rare and serious disorder almost exclusively seen in patients on dialysis or those with advanced chronic kidney disease (CKD) not on dialysis and is associated with very high mortality.

We present the case of a 50-year-old male with a background of end-stage renal disease (ESRD) compliant with dialysis, parathyroid adenoma, secondary hyperparathyroidism, and high body mass index (BMI). Whilst receiving 31 doses of intravenous sodium thiosulphate (STS) over an 11-week period, the patient underwent surgical debridement of multiple painful ulcerative lesions in his lower abdomen and left thigh and then subsequently a subtotal parathyroidectomy at 70 days from admission. Afterwards, he recovered from the surgery but became septic secondary to infection of calciphylaxis ulcers, later developed hospital-acquired pneumonia, and died with palliative care input.

Despite intravenous STS being the most commonly used drug in the treatment of calciphylaxis, it did not provide symptomatic relief or clinical improvement to the patient in this case. As there is no consensus on treatment duration for optimal response, this prompts future studies on the use of STS in addition to alternative treatment modalities of calciphylaxis.

## Introduction

Calciphylaxis is also known as calcific arteriopathy or calcific uremic arteriolopathy. This is an uncommon syndrome that affects approximately 1%-4% of patients with end-stage renal disease (ESRD) on dialysis, in rare cases during the late stage of chronic kidney disease (CKD stage 4 or 5), and following renal transplantation [1,2].

The pathogenesis is unknown [2]; however, it is thought to be caused by calcium deposition in small blood vessels within the superficial layer of the skin and fatty tissues [1]. This leads to obstruction within these vessels, resulting in poor blood supply to the affected tissues and thus causing ischemia and tissue death [1]. This presents as necrotic, non-healing ulcers with a predilection for the lower limbs and areas of the body rich in adipose tissue [3]. Factors such as hyperphosphatemia, hypercalcemia, secondary hyperparathyroidism, and hypercoagulability are thought to contribute to these skin lesions [4]. Calciphylaxis indicates a poor prognosis in patients with ESRD with a typical life expectancy of less than one year [3].

This case summary will discuss the diagnosis, treatment modality, and outcome of a 50-year-old male with a background of ESRD. He was compliant with dialysis for six years, had a body mass index (BMI) of 44.9 kg/m², and had a history of parathyroid adenoma and secondary hyperparathyroidism. He had no medical history of diabetes. He was not on warfarin or corticosteroid therapy. The therapeutic role of sodium thiosulphate (STS) used in his management will also be discussed.

## Case presentation

**Figure 1:**
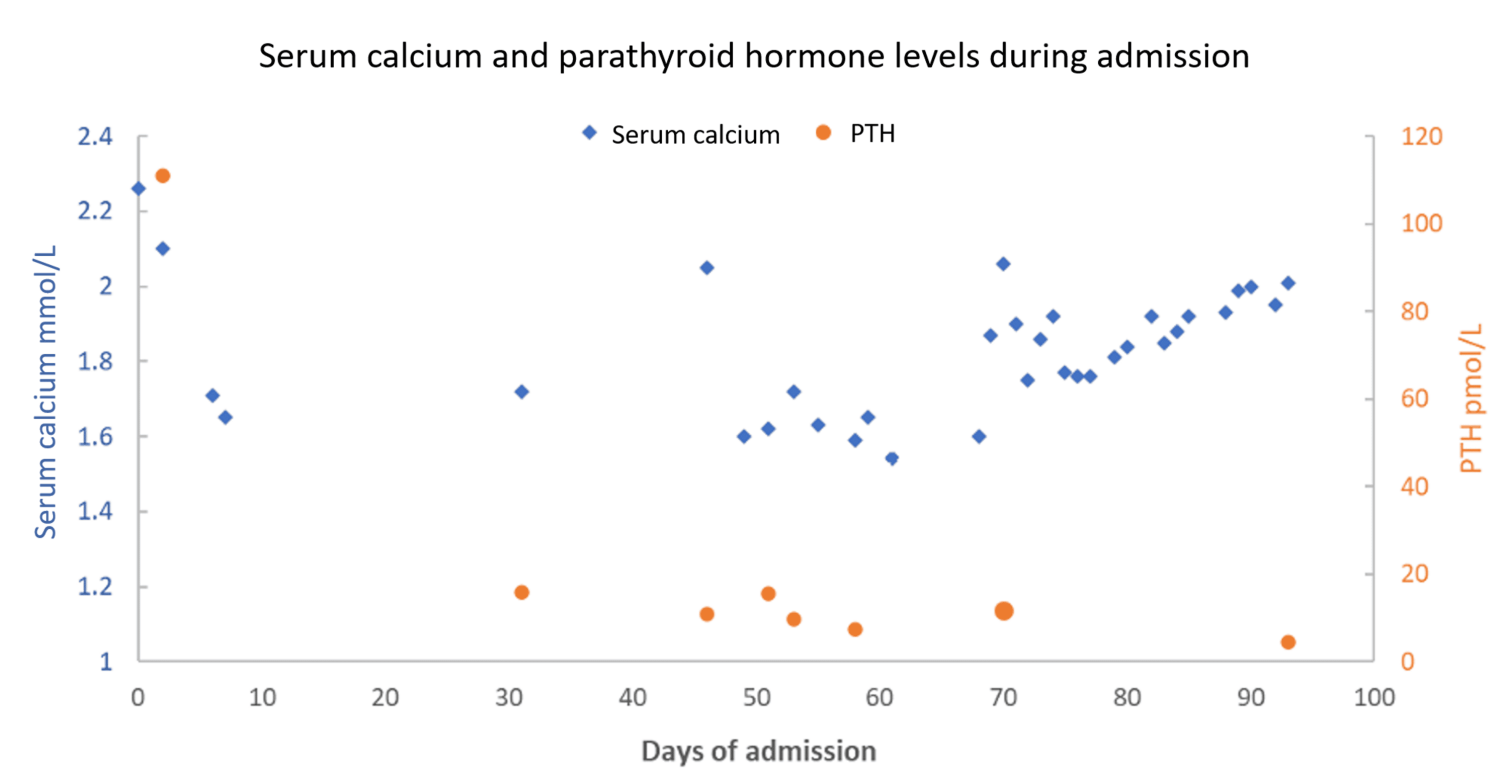
Trend of adjusted serum calcium during the 93-day admission of the 50-year-old male patient diagnosed with calciphylaxis Day 70 indicates the day of subtotal parathyroidectomy. PTH: parathyroid hormone

Here we present the case of a 50-year-old Caucasian male patient with ESRD secondary to hypertension who had thrice-weekly dialysis for the preceding six years in addition to secondary hyperparathyroidism with pre-admission blood tests: parathyroid hormone (PTH) 156 pmol/L, calcium 2.47 mmol/L, and phosphate 2.51 mmol/L (see Figure 1 for trend). He presented to the emergency department with two weeks of pain in his lower abdomen and left thigh. This pain and burning sensation were associated with the development of multiple ulcerative lesions in the aforementioned regions.

**Figure 2:**
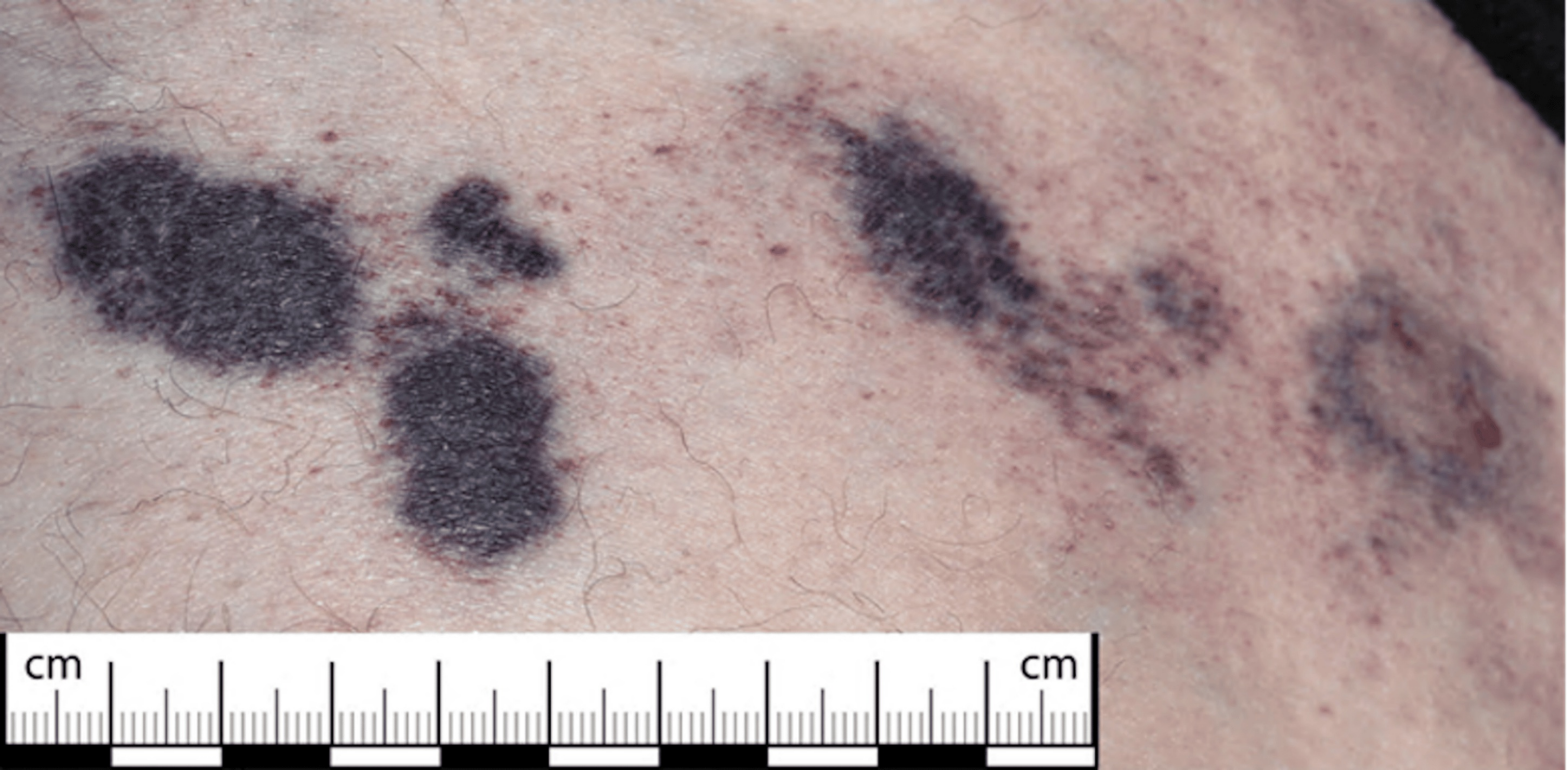
Indurated macules of the anterior-inferior aspect of abdominal wall with additional retiform lesions

**Figure 3:**
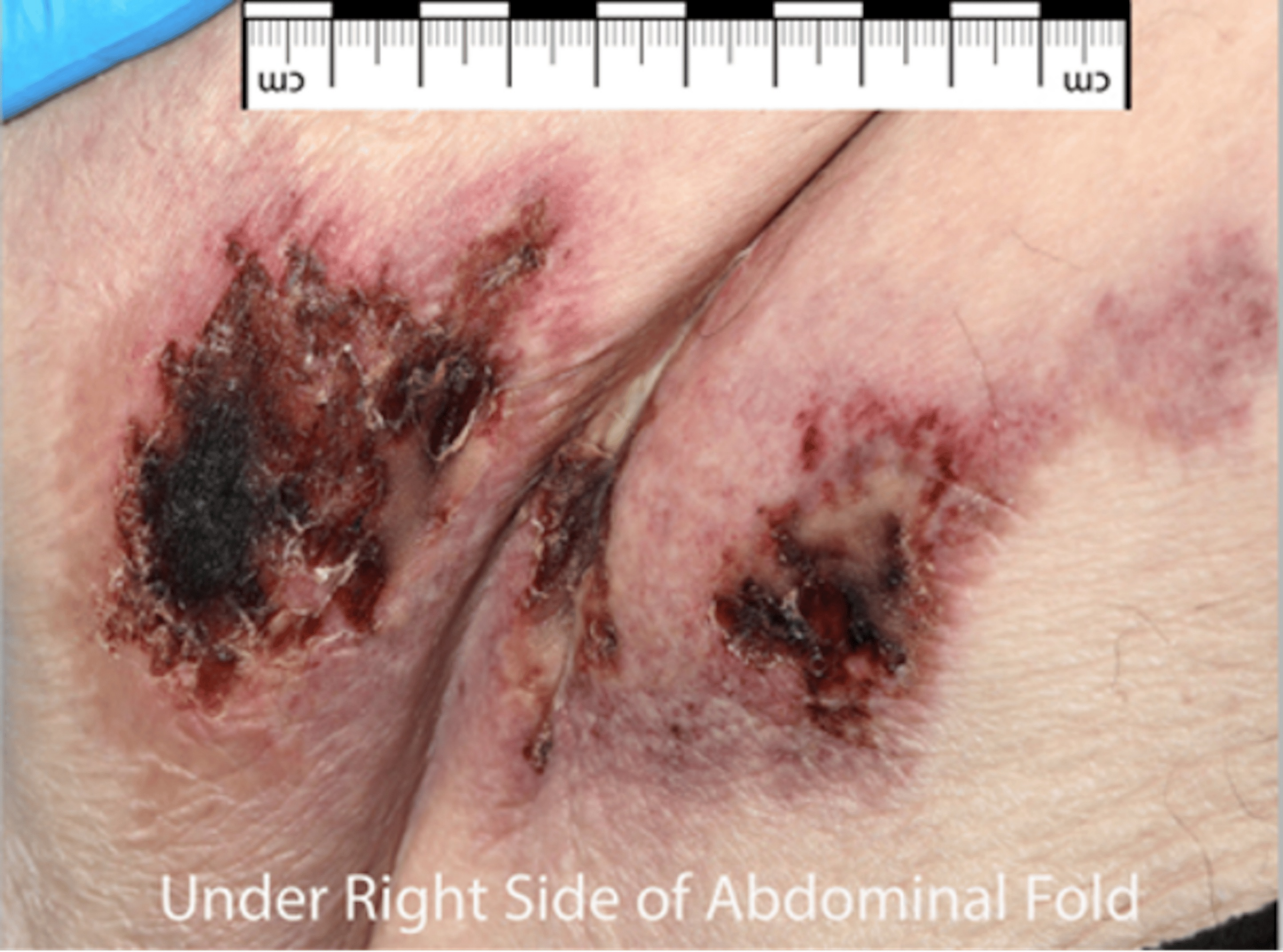
Superficial ulcerations with partial granulation tissue located to the lateral aspect of the abdomen with signs of secondary infection most marked at intertriginous areas

**Figure 4:**
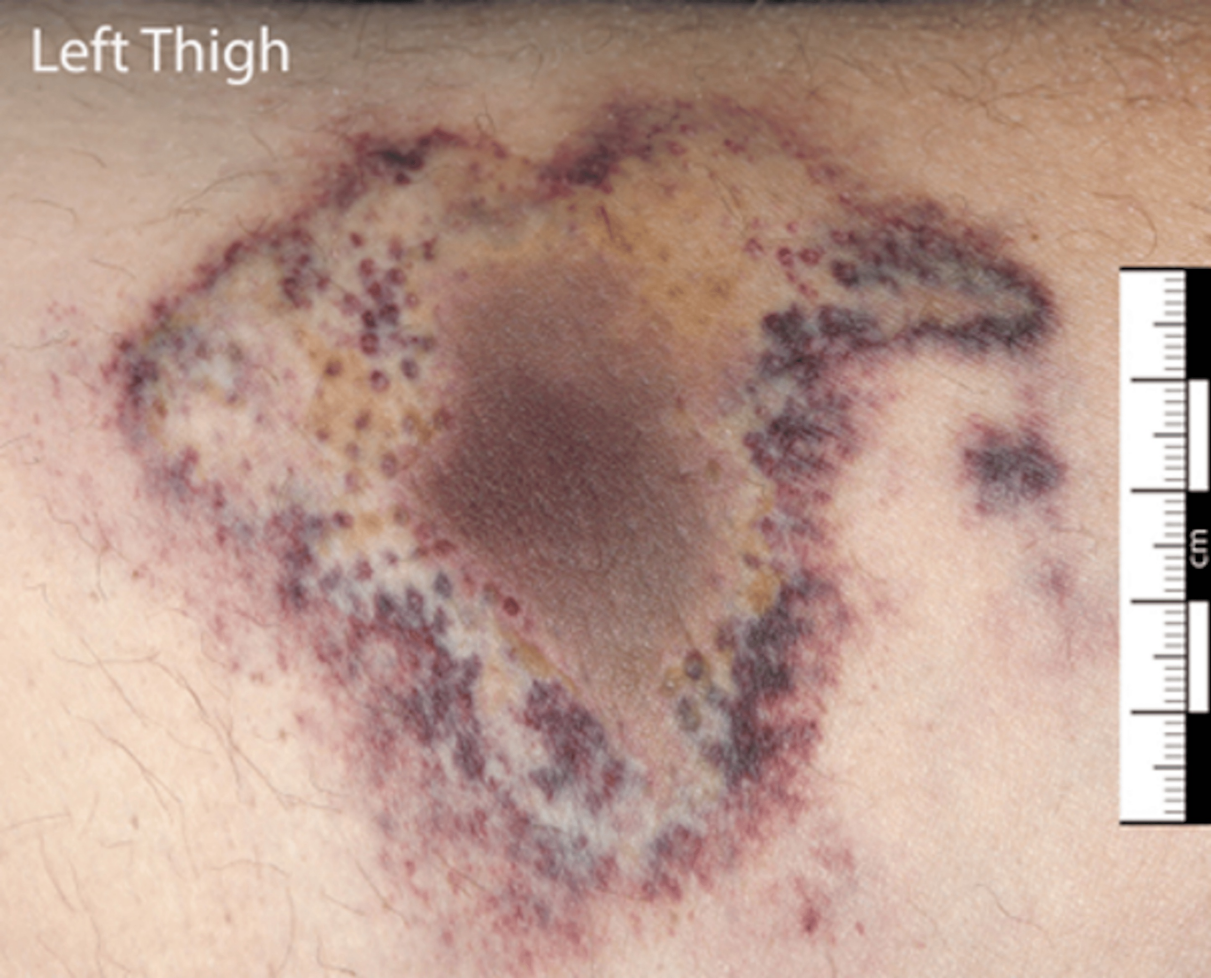
Indurated patch of the left thigh with an irregular outline of palisading pin-point macules

**Figure 5:**
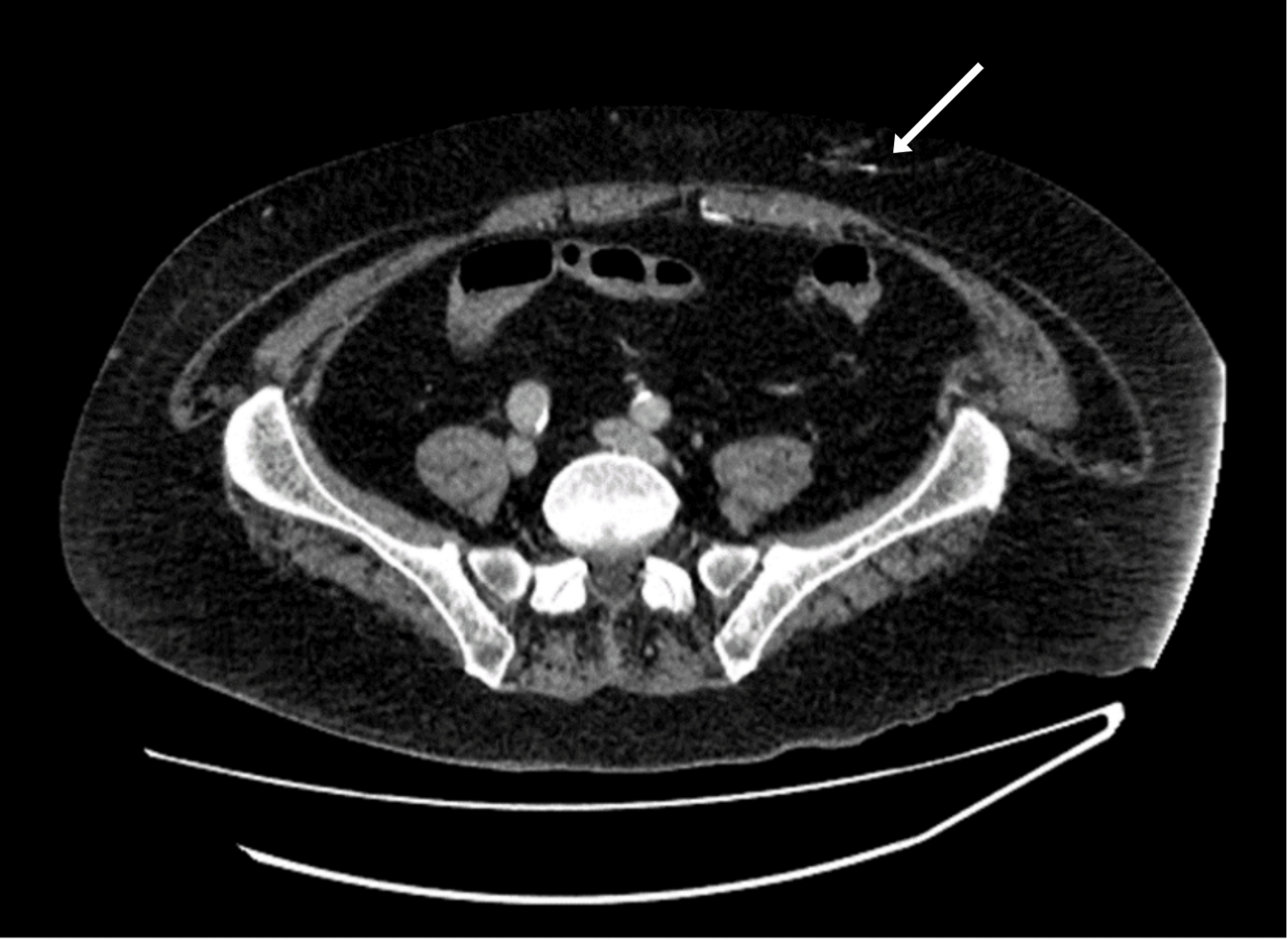
Venous phase contrast CT scan showing a localized area of inflammatory changes at the superficial anterior abdominal wall marked by an arrow

On examination, the lesions of the lower abdomen were of various morphology, ranging from 1 cm indurated retiform macules (Figure 2) to more extensive and multiple superficial ulcerations with signs of secondary infection at intertriginous areas (Figure 3). There was a 6 cm x 7.5 cm firm and tender patch of induration to the medial aspect of the left thigh (Figure 4). A CT scan of the abdomen and pelvis showed hyperdensities of the anterior abdominal wall consistent with active inflammation (Figure 5).

The patient was predisposed to the following risk factors of calciphylaxis: hyperparathyroidism, high body mass index, Caucasian ethnicity, and long dialysis vintage. However, he was not on warfarin therapy and had no problems adhering to his hemodialysis prescription. He had a background history of secondary hyperparathyroidism for more than six years, and despite taking the calcium mimetic cinacalcet, his calciphylaxis flared continuously.

The patient complained of intense pain that proved difficult to manage, which required up-titration of regular analgesia, including gabapentin, instant-release oxycodone, and transcutaneous fentanyl patches (12 mcg/hour). Additionally, he was offered fentanyl as part of patient-controlled analgesia (10 mcg IV mcg/ml 6 min lockout, 2 ml bolus). He frequently reported exacerbation of pain whilst receiving dialysis treatment and on mobilization.

Treatment

The patient’s wounds were surgically debrided alongside regular wound dressings. *Enterococcus*, *Pseudomonas*, and coliform species were cultured from the lesions with the secondary infection treated with meropenem in combination with clindamycin. Sodium thiosulphate 25 g was administered intravenously during the final hour of dialysis, which was conducted as four-hour sessions three times weekly. He received a total of 31 doses over an 11-week period.

He underwent subtotal parathyroidectomy 70 days after admission. Microscopy of the right superior and left inferior parathyroid glands showed features consistent with benign parathyroid adenoma. He subsequently recovered from the surgery.

Outcome

The patient later became septic secondary to infection of calciphylaxis ulcers. He required admission to the intensive care unit for 14 days and therefore was unable to receive STS therapy, as it could not be administered alongside hemofiltration. At day 93 of admission and 23 days post parathyroidectomy, his bone profile was: PTH 4.33 pmol/L, calcium 2.01 mmol/L, unadjusted albumin of 18 g/L, and phosphate 1.33 mmol/L. The patient then developed hospital-acquired pneumonia, and he died with palliative care input.

## Discussion

Calciphylaxis is a rare and serious disorder almost exclusively seen in dialysis and non-dialysis CKD stages 4 or 5 patients associated with very high mortality [1, 3]. It remains very rare, with approximately one case per year for every 600 dialysis patients in the UK [5]. The reported prevalence of calciphylaxis in dialysis patients ranges from 1% to 4%, with an annual incidence of around 0.04% [1-3].

Risk factors for the development of calciphylaxis in ESRD include Caucasian ethnicity, female sex, obesity with BMI >30 kg/m², diabetes, hypercalcemia, hyperphosphatemia, vitamin K deficiency, inflammatory conditions, hypoalbuminemia, hypercoagulable states (such as protein C and S deficiency and antiphospholipid syndrome), longer dialysis vintage, recurrent skin trauma, and medications including warfarin, calcium-based binders, vitamin D analogues, and corticosteroids [1,6,7]. In this case, the patient constituted most of the predisposing risk factors, which included obesity, Caucasian ethnicity, vitamin D analogues, and long vintage dialysis.

Lesion appearance varies with the time of presentation. In the early stage, violaceous, painful, plaque-like subcutaneous nodules, indurations, or livedo reticularis may develop, followed by ischemic or necrotic ulcers and painful eschars [3,7,8]. The affected sites most commonly involve the lower abdomen and proximal lower extremities. Less common lesion sites include the trunk, distal upper extremities, penis, breasts, and digits [3]. The lesions described in our case were in keeping with the typical distribution of calciphylaxis lesions [8]. Calciphylaxis has been shown to be difficult to diagnose; one retrospective study showed that approximately 77% were initially misdiagnosed as cellulitis (31%), unspecified skin infection (8%), and peripheral vascular disease (6.9%) [2,3,8,9]. Sepsis from infection of cutaneous wounds is the leading cause of death in patients with calciphylaxis [2,8].

Diagnosing calciphylaxis can be challenging, and there should be high suspicion in ESRD patients when they present with painful subcutaneous nodules, lesions, or non-healing ulcers, particularly in high adiposity, just as in this case. Clinical diagnosis is confirmed with a skin biopsy showing calcification, microthrombosis, and fibrointimal hyperplasia of skin arterioles [1,8]. However, skin biopsy may not be necessary among ESRD patients presenting with typical lesions, and biopsy itself is not without risk [3]. Punch or telescoping biopsy technique is recommended to avoid non-healing wounds and superimposed infection. Imaging, like X-rays, CT, and bone scans, can aid in diagnosis, as well as diagnosis of deep-seated infections [3].

Early diagnosis and treatment may minimize morbidity and mortality. A multidisciplinary approach is used regardless of the severity of the disease, including nephrology, dermatology, pain management, wound care, surgery, and nutrition [7]. Conventional therapy includes pain management, tight control of bone profile biochemistry (PTH, calcium, and phosphate), optimizing hemodialysis prescription, and excellent wound care [7].

Pain control is the most vital aspect of the management, as the excruciating nature of reported pain can lead to diminished quality of life [10,11]. Our patient had intense pain management involving pain specialist consultation. He did require ICU psychologist consultation due to the debilitating impact of the nature of pain from calciphylaxis.

The wound care team should be involved early, which includes selected dressings, chemical and surgical debridement, along with the administration of negative pressure wound therapy [11,12]. Our patient was regularly reviewed by a tissue viability nurse specialist, and he eventually underwent surgical debridement, requiring him to stay in the ICU for two weeks.

Sodium thiosulphate is the most used drug in the treatment of calciphylaxis, even though it is not a documented indication as per the British National Formulary [13-15]. Theoretically, STS interacts with calcium to form highly soluble complexes, which lowers calcium deposition in the blood vessels in addition to its vasodilatory and antioxidant properties, thereby achieving ischemic relief and pain reduction [12,16]. There is no consensus on optimal treatment duration, but empirically most patients receive a four-week trial of treatment and continue for a total of 12 weeks should there be benefits in pain control, wound healing, and bone profile biochemistry improvement [11,14]. Reported side effects include high anion gap metabolic acidosis, nausea, vomiting, volume overload, and hypocalcemia [16]. Despite completing 32 doses of STS over 11 weeks, and almost achieving empirical treatment, this did not elicit any significant clinical improvement, with further administration limited by his persistent hypotension secondary to sepsis, subsequent hospital-acquired infection, and use of hemofiltration during ICU admission. 

There have been several cases favouring STS [15,17-20]. However, our case seems to be different from some of the successful treatment cases of calciphylaxis. With regards to our patient’s declining condition at the end of STS therapy, it raises the question of whether intravenous STS provided any therapeutic benefits to the patient’s outcomes [21].

The prospective, randomized controlled trial, Evaluation of Cinacalcet Therapy to Lower Cardiovascular Events (EVOLVE trial), stated a 69%-75% reduction in the incidence of calciphylaxis with cinacalcet [22,23]. If PTH levels >300 pg/ml, then initiation of cinacalcet is considered to treat PTH-driven vascular calcification [22]. Our patient has been treated with cinacalcet while he developed uncontrolled secondary hyperparathyroidism but with no success. Even though parathyroidectomy is considered a treatment for calciphylaxis, sometimes parathyroidectomy has precipitated or worsened it by reducing bone turnover [24].

Concerning additional therapies, hyperbaric oxygen can be used in combination with STS to aid in calciphylaxis ulcer healing [25]. Its mechanism of action involves maximizing oxygenation to hypoxic tissues affected by calciphylaxis, allowing for collagen synthesis for tissue repair [26]. However, the association between the number of hyperbaric oxygen therapy sessions and wound healing remains disputed [27].

Regarding other emerging treatments, some advocate that rheopheresis is effective for treatment despite an unclear mechanism of action [28]. Low-dose infusions of tissue-type plasminogen activator may be used for treatment-resistant calciphylaxis patients with hypercoagulable states, and again the mechanism of action was unclear [29]. Some case reports have described sterile maggot therapy with larvae of the greenbottle fly, *Lucilia sericata* [30]. As prevention is better than cure, clinicians should consider the optimal management of modifiable risk factors in patients at high risk of developing calciphylaxis, such as strict control of bone profile biochemistry in those with tertiary hyperthyroidism and weight loss in those with high body mass index.

From the outlook of this case, newer treatment options should be explored by conducting larger clinical trials [16,31]. Treatment should be initiated as soon as the diagnosis of calciphylaxis is made as per current recommendations, literature, and the latest case reports [14,15,17]. Despite the maximal therapy for calciphylaxis, we still see a very poor prognosis with more than 50% mortality at one year and more than 80% if there is ulceration [4,7].

## Conclusions

Diagnosing calciphylaxis is challenging, but there should be a higher index of suspicion in patients with CKD stages 4 or 5, on dialysis or post renal transplant with identified risk factors and painful cutaneous lesions. In this case, treatment with intravenous sodium thiosulfate did not yield any clinical improvement, highlighting the need for alternative, more effective therapies.
